# Impact of a Multifactorial Educational Training on the Management of Preterm Infants in the Central-Eastern European Region

**DOI:** 10.3389/fped.2021.700226

**Published:** 2021-08-30

**Authors:** Philipp Steinbauer, Katrin Klebermass-Schrehof, Francesco Cardona, Katharina Bibl, Tobias Werther, Monika Olischar, Georg Schmölzer, Angelika Berger, Michael Wagner

**Affiliations:** ^1^Division of Neonatology, Pediatric Intensive Care and Neuropediatrics, Department of Pediatrics, Comprehensive Center for Pediatrics Medical University of Vienna, Vienna, Austria; ^2^Centre for the Studies of Asphyxia and Resuscitation, Neonatal Research Unit, Royal Alexandra Hospital, Edmonton, AB, Canada; ^3^Division of Neonatology, Department of Pediatrics, University of Alberta, Edmonton, AB, Canada

**Keywords:** less invasive surfactant administration, extremely preterm infants, lung development, outcome, delivery room management, educational training concept

## Abstract

**Background:** Differences in management and outcomes of extremely preterm infants have been reported across European countries. Implementation of standardized guidelines and interventions within existing neonatal care facilities can improve outcomes of extremely preterm infants. This study evaluated whether a multifactorial educational training (MET) course in Vienna focusing on the management of extremely preterm infants had an impact on the management of extremely preterm infants in Central-Eastern European (CEE) countries.

**Methods:** Physicians and nurses from different hospitals in CEE countries participated in a two-day MET in Vienna, Austria with theoretical lectures, bedside teaching, and simulation trainings. In order to evaluate the benefit of the workshops, participants had to complete pre- and post-workshop questionnaires, as well as follow-up questionnaires three and twelve months after the MET.

**Results:** 162 participants from 15 CEE countries completed the two-day MET at our department. Less invasive surfactant administration (LISA) was only used by 39% (63/162) of the participants. After the MET, 80% (122/152) were planning to introduce LISA, and 66% (101/152) were planning to introduce regular simulation training, which was statistically significantly increased three and twelve months after the MET. Thirty-six percent and 57% of the participants self-reported improved outcomes three and twelve months after the MET, respectively.

**Conclusion:** Our standardized training in Vienna promoted the implementation of different perinatal concepts including postnatal respiratory management using LISA as well as regular simulation trainings at the participants' home departments. Moreover, our MET contributed to dissemination of guidelines, promoted best-practice, and improved self-reported outcomes.

## Introduction

The burden of preterm birth extends globally and contributes significantly to neonatal morbidity and mortality (1). Advances in obstetrics and neonatal care over the last decades have significantly increased survival of preterm infants (2). However, differences in management and outcomes have been reported within and across European countries (3, 4). Although perinatal morbidity and mortality are constantly decreasing in Central-Eastern-European (CEE) countries (5, 6), some of these countries are still facing higher challenges with regards to patient outcomes compared to others (5).

Variations in outcomes between European countries may be associated with differences in maternal demographic and socioeconomic characteristics (7), as well as differences in quality of antenatal and perinatal care (8–10). There is a lack of data about the causes of neonatal deaths in CEE countries. However, it has been estimated that 80% of neonatal deaths in CEE countries are caused by birth asphyxia, severe infections, and complications of prematurity (11). Implementation of standardized guidelines and interventions within existing neonatal care facilities can improve mortality of newborns in need of intensive care (12–14).

We invited physicians and nurses from CEE countries to participate in a two-day multifactorial educational training (MET) course to discuss standardized interventions (i.e., delivery room management, less invasive surfactant administration (LISA), strategies to avoid mechanical ventilation, principles of developmental care, and simulation training of neonatal emergency situations) to improve neonatal outcomes.

We aimed to evaluate whether MET facilitates the implementation of LISA, a change of perinatal management, and regular simulation trainings at the participants' home departments. Furthermore, we evaluated if the MET course would result in management changes at the participants' home institution as well as improved self-reported outcomes.

## Materials and Methods

### Participants and Setting

**Figure 1:**
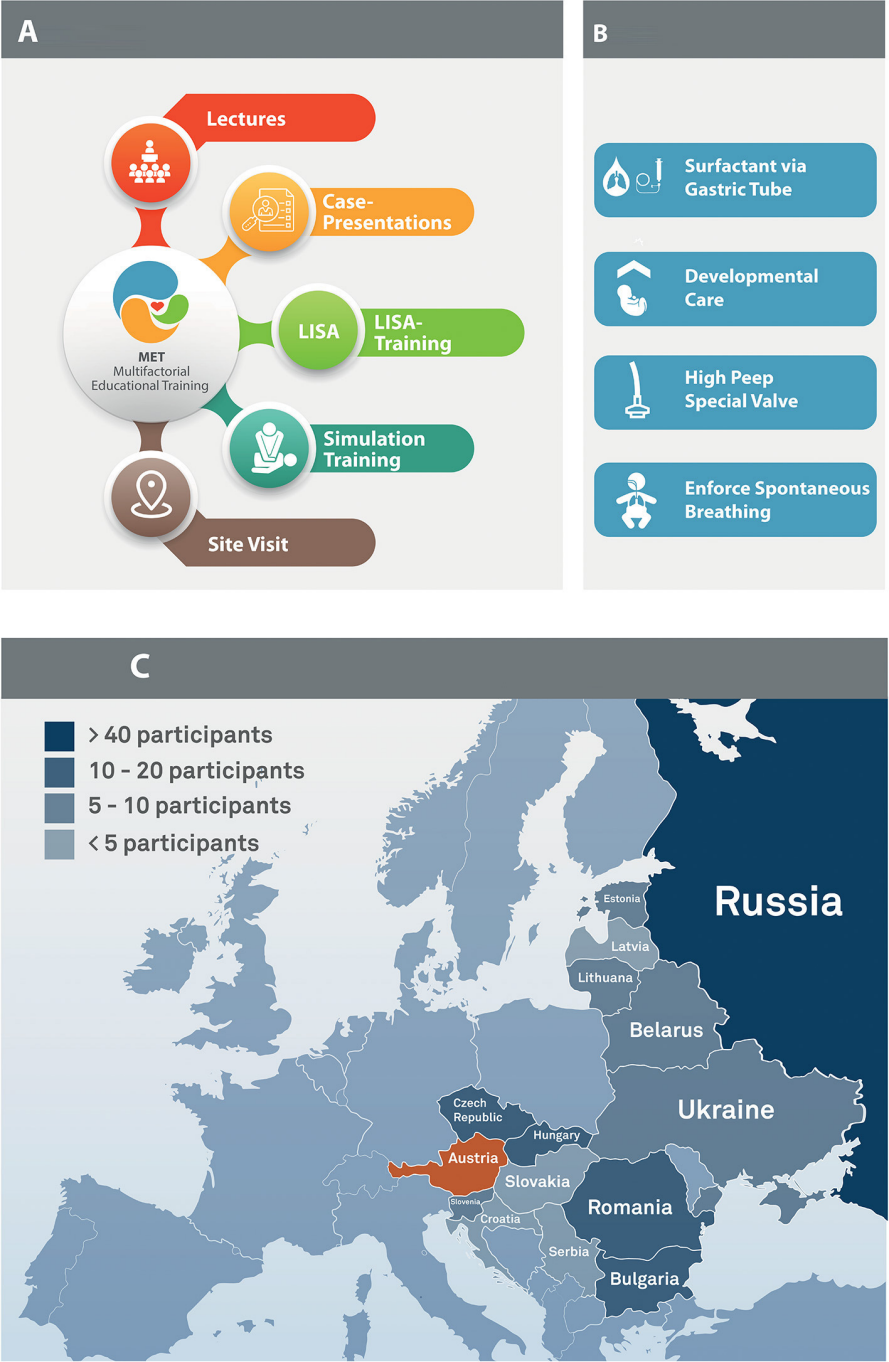
**(A)** Overview of the multifactorial educational training, **(B)** Overview of “The Viennese Concept”, **(C)** Overview of all participating countries. Uzbekistan not in the figure.

Between 2014 and 2018, physicians and nurses from several CEE hospitals were invited to participate in a two-day MET at the Department of Pediatrics at the Medical University of Vienna, Austria. Our MET was structured like a collaborative quality improvement workshop with the ultimate goal to share theoretical and practical knowledge on different surfactant application approaches (with a focus on LISA), delivery room management, neonatal emergencies, and postnatal management of extremely premature infants. Throughout the MET, participants attended theoretical lectures, LISA and simulation trainings, as well as bedside teaching in our neonatal intensive care unit (Figure 1A). Participants completed a pre- and post-MET course questionnaire and an e-mail follow-up-questionnaire three and 12 months after the MET course. Further, participants had the possibility to stay in contact with our team via e-mail to help with any questions regarding management or changes of management in extremely preterm babies. The study was exempt from ethics review by the local ethics committee.

### The MET Course

#### Lectures and Cases

The course started with state-of-the-art lectures and case presentations about delivery room and developmental care principles, including approaches to minimally invasive respiratory management, infections, and patient safety. Furthermore, “The Viennese Concept”, which represents the local approach to postnatal management of extremely premature infants, was presented. The “Viennese Concept” (Figure 1B) includes stabilization on high-flow CPAP (via a special valve), LISA via a small endotracheal catheter during spontaneous breathing of the infant (prophylactic surfactant administration, and avoidance of mechanical ventilation) (15). Particular attention is given to reducing stress (no light, reducing noise, nesting/facilitated tucking, non-pharmacological analgesia).

#### LISA Training

After a demonstration, participants practiced the LISA technique with continuous feedback from the instructors using the PremieHal (Gaumard Scientific, Miami, Florida) or Paul (SIMCharacters GmbH, Vienna, Austria) manikin.

#### High-Fidelity Simulation Training

Participants received a lecture about the general principles of simulation trainings, human factors, crisis resource management, teamwork, and communication. Afterwards, attendees used the Paul and SimNewB (Laerdal, Stavanger, Norway) manikins to practice delivery room management including szenarios on a i) term infant with meconium aspiration syndrome and ii) mechanically ventilated preterm infant with a sudden decrease in oxygen saturation due to an occluded endotracheal tube. The scenarios were video recorded using SIMStation (SIMStation GmbH, Vienna Austria). After each simulation, a structured debriefing video analysis with focus on human factors including teamwork and communication was performed.

#### Site Visit

Participants were guided through the department's four neonatal intensive and intermediate care units (NICU, NIMCU) and thus gained insight into daily clinical work and got to know the structural, logistical, and IT-solutions in our wards. Daily clinical work in Vienna and similarities as well as differences to the participant's home institutions were discussed. Furthermore, there were discussion rounds on how to implement these new ideas and concepts in NICU teams at their respective home institutions.

#### Questionnaires

Questionnaires were used to evaluate the benefit of our MET course. The pre-MET questionnaire included demographic data of participants, home institutional guidelines, current clinical standards as well as teamwork and communication during LISA/emergency situations in the home institution. Post-MET questionnaires assessed the participants' experience with the MET course and the individual learning effect. The follow-up questionnaires aimed to determine whether the MET course impacted the participants' approach to LISA, neonatal emergencies, teamwork, and communication, whether Viennese concepts and standards were introduced in the participants' home institution and whether the outcome and/or patient safety in the participant's home institution improved following the MET course in Vienna.

### Statistical Analysis

Collected data were analyzed retrospectively and descriptively. Categorical variables are presented in absolute frequencies and percentages. McNemar's test was used to compare pre and post MET questionnaires. Data analysis was performed using SPSS statistics for Mac, version 24 (IBM, New York City, New York). The level of significance was set at *P* < 0.05 (two-tailed).

## Results

Between October 2014 and November 2018, 162 health care providers from 15 CEE countries (Figure 1C) finished the two-day MET at our department (Table 1). Of the 152 participating physicians, 72% were head (72/152) or consultants (43/152) with more than ten years of work experience. In addition ten nurses participated in our MET. All participants (100%; 162/162) completed the pre-MET questionnaire and 94% (152/162) the post-MET questionnaire. Three-month and 12-month follow-up questionnaires were completed by 34% (55/162) and 19% (31/162) of all participants, respectively (Table 1).

**Table 1:** Demographic characteristics of healthcare professionals (*n* = 162).

**Characteristics**	***n*** **(%)**
**Gender**	
Female	108 (67)
Male	54 (33)
**Age distribution**	
<30	9 (6)
30–44	81 (50)
45–54	54 (33)
>54	18 (11)
**Professional status**	
**Physician**	152 (94)
Head	72 (45)
Consultant	43 (27)
Fellow	25 (15)
Resident	12 (7)
Nurse	10 (6)
**Number of participants**	
All participants	162 (100)
Belarus	5 (3)
Bulgaria	15 (9)
Croatia	4 (2)
Czech Republic	17 (11)
Estonia	5 (3)
Hungary	12 (8)
Latvia	4 (2)
Lithuania	5 (3)
Romania	13 (8)
Russia	55 (34)
Serbia	4 (2)
Slovakia	4 (2)
Slovenia	9 (6)
Ukraine	7 (5)
Uzbekistan	3 (2)
**Number of Centers**	
All centers	125 (100)
Belarus	3 (2)
Bulgaria	14 (11)
Croatia	4 (3)
Czech Republic	6 (5)
Estonia	4 (3)
Hungary	12 (10)
Latvia	4 (3)
Lithuania	2 (2)
Romania	10 (8)
Russia	49 (39)
Serbia	3 (2)
Slovakia	4 (3)
Slovenia	2 (2)
Ukraine	7 (6)
Uzbekistan	1 (1)

### Pre-MET Questionnaire and Standard of Care

Satisfaction with the MET was high among participants. Particularly, 95% of the participants (144/152) who completed the post-MET questionnaire reported a “very good experience”. Simulation training (59%, 90/152) and lectures/cases (59%, 89/152) were the most popular parts of the educational session, followed by demonstration of LISA (52%; 79/152).

Of the 162 participants who completed the pre-MET questionnaire, 65% (105/162) reported a standardized protocol for surfactant administration in their home department. LISA was only used by 39% (63/162) of the participants, INSURE was used by 57% (93/162) and tube surfactant with mechanical ventilation and extubation when ready was reported by 50% (81/162) (Table 2). While LISA was used by 8% (13/162) of the participants as the only method to administer surfactant, 16% (26/162) of the participants considered INSURE as the best way to deliver surfactant during perinatal management. Furthermore, 16% (26/162) of the participants stated that they exclusively used intubation and mechanical ventilation for surfactant administration in the delivery room.

**Table 2:** Mode of surfactant application in the delivery room prior to the workshops (*n* = 162).

**Parameter**	***n*** **(%)**
**Standardized protocol**	
yes	105 (65)
no	57 (35)
**Surfactant delivery**	
LISA	63 (39)
INSURE	93 (57)
Mechanical ventilation	81 (50)
**Premedication use**	57 (35)
Regularly	36 (63)
Sometimes	21 (37)
**Premedication - substances**	
Opioids	37 (65)
Benzodiazepines	22 (39)
Propofol	8 (14)
Ketamine	4 (7)
Thiopental	3 (5)
Muscle relaxants	3 (5)
Atropine	3 (5)
**Adverse Events during surfactant application**	147 (91)
Decrease of Saturation	130 (88)
Bradycardia	60 (41)
Regurgitation	54 (37)
Apnea	28 (19)
Endotracheal obstruction	4 (3)

### Impact of MET on the Change of Local Management

**Figure 2:**
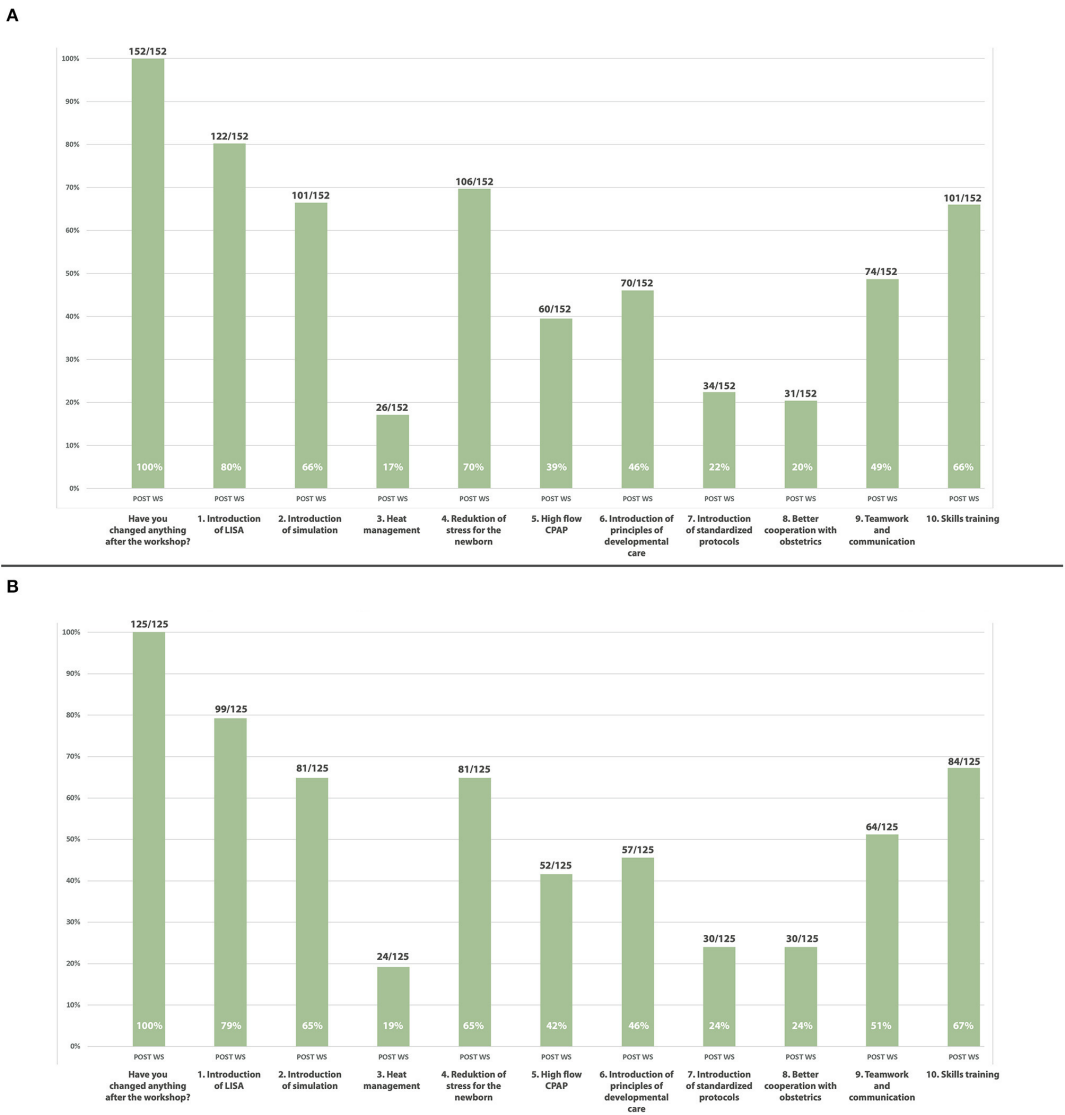
**(A)** Overview of responses post multifactorial educational training (*n* = 152), **(B)** Overview of responses according to each center after the multifactorial educational training (*n* = 125).

After the MET, all participants who completed the post-training questionnaire (152/152) stated that they planned to implement some of the standards and concepts learned during the MET at their home institution. More specifically, 80% (122/152) were planning to introduce LISA, 66% (101/152) were planning to introduce regular simulation trainings, 70% (106/152) wanted to pay more attention on reducing stress of the preterm baby, and 49% (74/152) were prepared to focus more on better teamwork and communication (Figure 2A). An overview of responses according to each center after the MET is provided in Figure 2B.

### Impact on the Change of Management Three and Twelve Months After the MET

**Figure 3:**
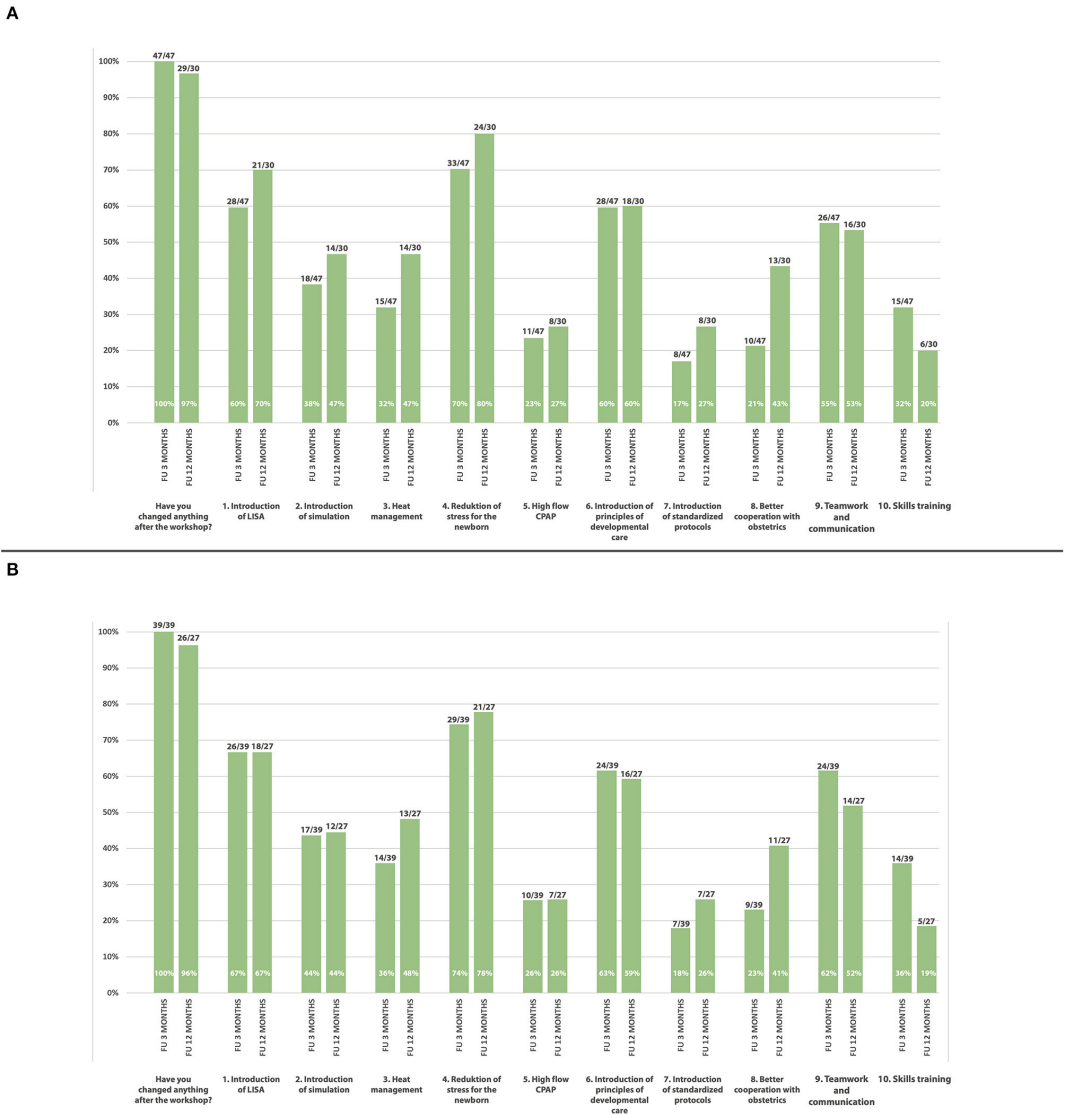
**(A)** Overview of responses of each participant three (*n* = 47) and twelve (*n* = 30) months after the multifactorial educational training, **(B)** Overview of responses according to each center three (*n* = 39) and twelve (*n* = 27) months after the multifactorial educational training.

Forty-seven of 162 (29%) participants completed the follow-up questionnaire three months after the MET. Thirty participants (19%) completed the follow-up questionnaire after twelve months. One hundred percent (47/47) and 97% (29/30) of the respondents had changed anything in their clinical management three months and twelve months after the MET, respectively. Of those participants who conducted changes, 70% after three (80% after twelve) months had paid more attention on reducing stress of the newborn, 38% (47%) had introduced regular simulation trainings, 60% (60%) had introduced new principals of developmental care, 60% (70%) had introduced LISA in their department, and 55% (53%) had focused more on teamwork and communication, 32% (47%) had introduced better heat management, 23% (27%) had implemented high-flow CPAP, and 17% (27%) had introduced standardized protocols three and twelve months after the MET, respectively (Figure 3A).

An overview of responses according to each center after the MET is provided in Figure 3B.

**Figure 4:**
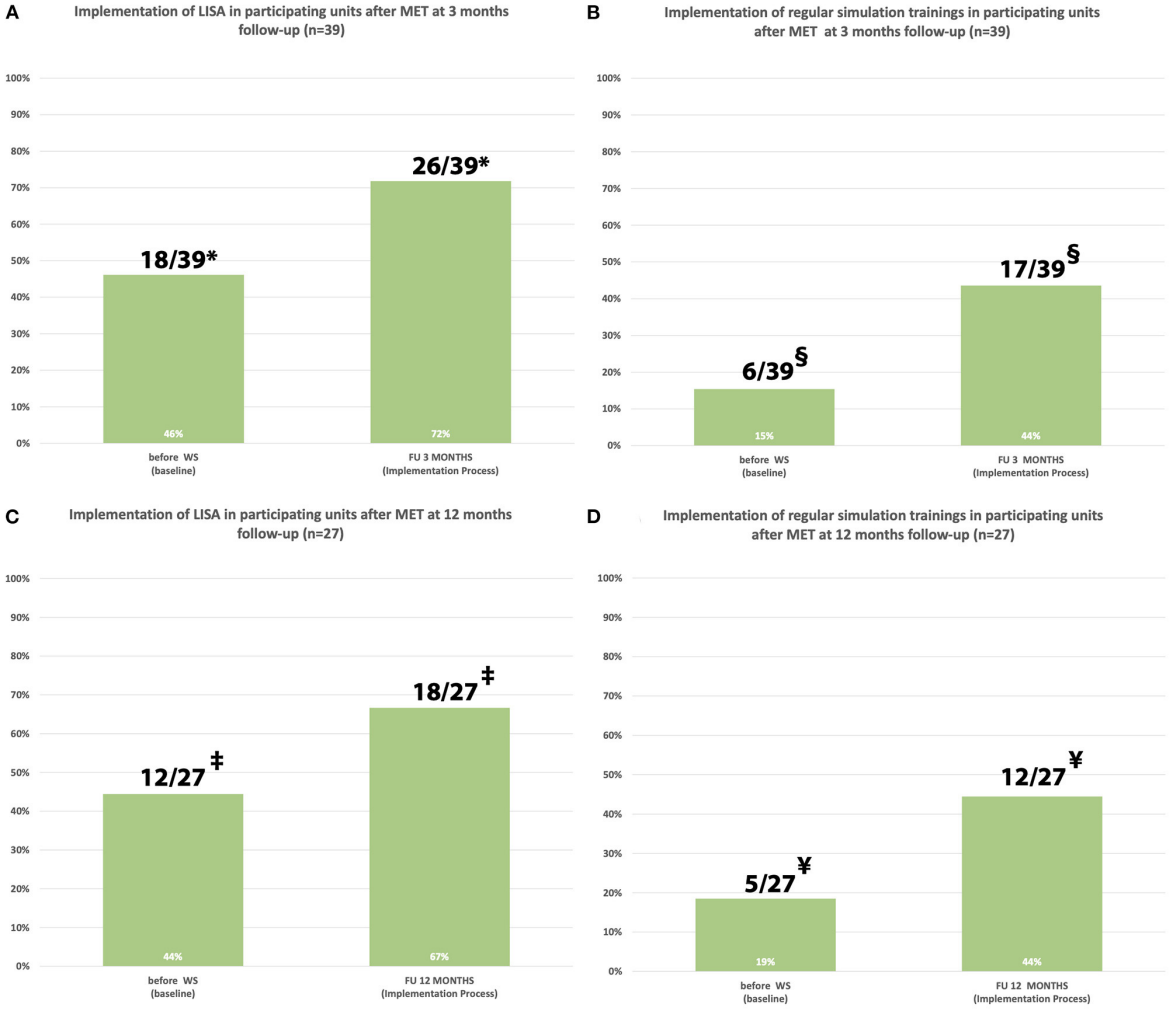
**(A–D)** Implementation of LISA and regular simulation trainings in the home department at three and twelve months after the multifactorial educational training according to participating units. *Statistically significant, *p* = 0.013; ‡statistically significant, *p* = 0.021; §statistically significant, *p* = 0.03; ¥statistically significant, *p* = 0.023.

There was a statistically significant increase of participants who used LISA regularly three and twelve months after when compared to before the MET (*p* = 0.013 and *p* = 0.021, respectively) (Figures 4A,C). In addition, three and twelve months after the MET there was a significant increase in the proportion of participants who started regular simulation trainings in the home departments when compared with simulation training activities before the MET (*p* = 0.03 vs. *p* = 0.023, respectively) (Figures 4B,D).

Many participants reported difficulties with implementation of change processes in the home institution, such as shortage of physicians and nurses as main obstacle for successful change processes and reported structural difficulties as main obstacle for a change. Furthermore, some participants assumed that other physicians or nurses in their home department were reluctant to change.

### Impact on Patient Outcomes Three and Twelve Months After the MET

**Figure 5:**
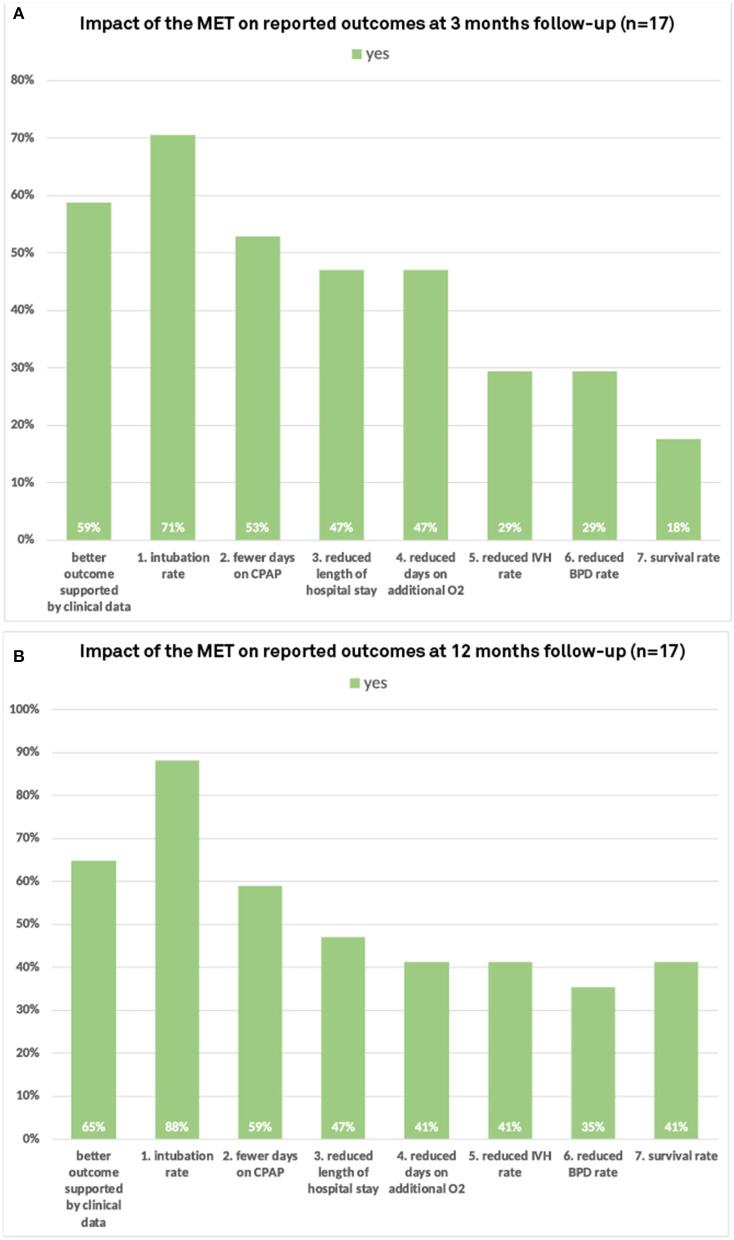
Impact of the multifactorial educational training on observed patient outcomes at **(A)** three and **(B)** twelve months follow-up.

Thirty-six percent (17/47) and 57% (17/30) of the participants reported improved patient outcomes three and twelve months after the MET, respectively. Of all participants who reported improved outcomes, 71% (88%) observed a decrease in intubation rates and fewer days on mechanical ventilation, 52% (59%) observed fewer days on CPAP, 47% (47%) observed reduced lengths of hospital stay, 47% (41%) reported reduced days on additional oxygen, and 29% (41%) reported reduced rates of IVH and bronchopulmonary dysplasia 29 (35%) three and twelve months after the MET, respectively (Figures 5A,B). In particular, 59% (65%) of these participants stated that they were able to verify improved outcomes with statistical data three and twelve months after the MET, respectively.

## Discussion

To our knowledge, this is the first study to examine the impact of an international MET covering postnatal respiratory management, surfactant administration, principles of developmental care, and simulation training on the change of local management at the participants home departments. Our standardized MET significantly contributed to a re-evaluation of local management including a potential implementation of LISA and regular simulation trainings at the participants home departments as stated by participants in the questionnaires three and twelve months after the MET. Moreover, participants reported that they subjectively observed improved clinical outcomes after the implementation of LISA and other concepts of the MET at their home department.

In 2008, we modified a method previously described by Kribs et al. (16) and established a standardized, less invasive postnatal respiratory management “Viennese” protocol including early high flow CPAP and surfactant administration in spontaneously breathing infants via a thin catheter placed into the trachea (15). According to the European (17) and British (18) RDS guidelines, LISA is the preferred method of surfactant administration in spontaneously breathing infants including the need for regular training session as supported by The European Foundation for the Care of Newborn Infants (EFCNI) (19). Vento and colleagues provided an extensive overview of different methods to deliver surfactant and gave insights on how to organize and structure training programs for thin catheter surfactant delivery (20). However, literature (21) and our personal experience showed that LISA was not widely adopted across CEE countries. Knowing that LISA can help to improve outcomes (22), we wanted to show other centers that have limited or no experience with LISA, how LISA and our “Viennese concept” can be adopted at their home departments for quality improvement.

Hence, we invited neonatologists from CEE countries to a standardized two-day MET in Vienna in order to share theoretical and practical experience with LISA and other delivery room concepts, with the ultimate goal to facilitate the implementation of those concepts at the participants' home departments. Neonatal networks encompassing regions and countries offer the perfect platform for implementing quality improvement projects in order to exchange local guidelines and knowledge (23). An active approach to research dissemination such as workshops are both feasible and cost-effective and can help to effectively translate research evidence into clinical practice (24). As a result of our MET, the implementation of LISA increased from 43 to 60% three months after our MET and from 43 to 70% twelve months after our MET in all participating centers. Participants who implemented LISA after our workshop observed lower intubation rates with the LISA concept. This is consistent with data of popular RCTs (25, 26) and observational studies (15, 27, 28), reporting a decrease in need for intubation after LISA.

Recent surveys revealed an increased use of LISA within European countries with a rapid growth from less than 10% in 2010 up to 52% in 2015 of all units assessed (21). The initial sparse use of LISA was probably related to the small number of published studies and limited data until 2015. Moreover, a wide variation of LISA use was found in Nordic countries ranging from 9 to 100% of all units assessed (29). When compared to western European centers, LISA was not widely adopted in CEE countries. This situation illustrates that the up-take of new practices and guidelines significantly varies between different geographical areas. Since LISA was developed in Germany (16) it is not surprising that it was initially almost exclusively used in Germany before our unit adopted and modified the method (15). While before our MET only 43% (20/47) of all CEE participants used LISA, after our MET the number increased to 60% (28/47). Consequently, our MET contributed to the guideline dissemination within CEE countries. Moreover, we introduced not only a simple workshop, but a multifactorial educational session for all participants, which was new for this topic. Our MET is a good example of how it may work to adopt existing guidelines, successfully implement them at the local unit, and thus improve clinical outcomes of premature infants (15). Consequently, the intention of our MET was to share knowledge, practice, and outcome data of LISA with centers not experienced in this method. Furthermore, we aimed to assist units with the implementation process at their home department later on. Our MET resulted in management changes at the participants' home institution as well as improved self-reported outcomes.

## Limitations

Our study has several limitations. First, the design of the study was only observational. Second, as with other surveys, responder bias could not be ruled out since answers may not be representative for the unit practices. Third, the outcomes of this study were only based on subjective questionnaires responses and the response rate of follow-up questionnaires was low and the number of responses varied between countries. We speculate that the reason for the low response rate was that we sent the follow-up questionnaires as PDF files via e-mail and asked for scanning them to send it back rather than using an online survey tool. Further, some participants were from the same center, which potentially distorted parts of our results. Consequently, we also provided data of participants according to their affiliation. Nevertheless, we presented a broad overview on the change of management after our MET course across CEE countries. Fourth, because of the study design we were not able to exclude the possibility that the improvement in outcomes was caused by other influencing factors. In addition, self-reported improvement of outcomes was only assessed by a follow-up questionnaire three and twelve months after the MET and the study was not designed to assess clinical outcomes. We are aware that self-reported observed outcomes which are not supported by actual objective data are prone to bias.

## Conclusion

Our intervention in the format of a two-day MET course, including our standardized postnatal respiratory management protocol with LISA and simulation-based trainings, promoted the implementation of different perinatal concepts including postnatal respiratory management with LISA as well as regular simulation trainings at the participants home departments. Moreover, our MET contributed to dissemination of guidelines, promoted best-practice, and improved self-reported clinical outcomes.

## Data Availability Statement

The raw data supporting the conclusions of this article will be made available by the authors, without undue reservation.

## Ethics Statement

Ethical review and approval was not required for the study on human participants in accordance with the local legislation and institutional requirements. The patients/participants provided their written informed consent to participate in this study.

## Author Contributions

PS, MO, GS, and MW conceptualized and designed the study, drafted the initial manuscript, and reviewed and revised the manuscript. KK-S, FC, KB, TW, and AB helped with data collection, the analysis process, and reviewed and revised the manuscript. All authors approved the final manuscript as submitted and agree to be accountable for all aspects of the work.

## Conflict of Interest

The authors declare that the research was conducted in the absence of any commercial or financial relationships that could be construed as a potential conflict of interest.

## Publisher's Note

All claims expressed in this article are solely those of the authors and do not necessarily represent those of their affiliated organizations, or those of the publisher, the editors and the reviewers. Any product that may be evaluated in this article, or claim that may be made by its manufacturer, is not guaranteed or endorsed by the publisher.
